# Mimotope-Based Vaccines of *Leishmania infantum* Antigens and Their Protective Efficacy against Visceral Leishmaniasis

**DOI:** 10.1371/journal.pone.0110014

**Published:** 2014-10-15

**Authors:** Lourena Emanuele Costa, Luiz Ricardo Goulart, Nathália Cristina de Jesus Pereira, Mayara Ingrid Sousa Lima, Mariana Costa Duarte, Vivian Tamietti Martins, Paula Sousa Lage, Daniel Menezes-Souza, Tatiana Gomes Ribeiro, Maria Norma Melo, Ana Paula Fernandes, Manuel Soto, Carlos Alberto Pereira Tavares, Miguel Angel Chávez-Fumagalli, Eduardo Antonio Ferraz Coelho

**Affiliations:** 1 Programa de Pós-Graduação em Ciências da Saúde: Infectologia e Medicina Tropical, Faculdade de Medicina, Universidade Federal de Minas Gerais, Belo Horizonte, Minas Gerais, Brazil; 2 Instituto de Genética e Bioquímica, Universidade Federal de Uberlândia, Uberlândia, Minas Gerais, Brazil; 3 Department of Medical Microbiology and Immunology, University of California Davis, Davis, CA, United States of America; 4 Departamento de Bioquímica e Imunologia, Instituto de Ciências Biológicas, Universidade Federal de Minas Gerais, Belo Horizonte, Minas Gerais, Brazil; 5 Departamento de Parasitologia, Instituto de Ciências Biológicas, Universidade Federal de Minas Gerais, Belo Horizonte, Minas Gerais, Brazil; 6 Programa de Pós-Graduação em Ciências Farmacêuticas, Faculdade de Farmácia, Universidade Federal de Minas Gerais, Belo Horizonte, Minas Gerais, Brazil; 7 Departamento de Análises Clínicas e Toxicológicas, Faculdade de Farmácia, Universidade Federal de Minas Gerais, Belo Horizonte, Minas Gerais, Brazil; 8 Centro de Biología Molecular Severo Ochoa, CSIC-UAM, Departamento de Biología Molecular, Universidad Autónoma de Madrid, Madrid, Spain; 9 Departamento de Patologia Clínica, COLTEC, Universidade Federal de Minas Gerais, Belo Horizonte, Minas Gerais, Brazil; Federal University of São Paulo, Brazil

## Abstract

**Background:**

The development of cost-effective prophylactic strategies to prevent leishmaniasis has become a high-priority. The present study has used the phage display technology to identify new immunogens, which were evaluated as vaccines in the murine model of visceral leishmaniasis (VL). Epitope-based immunogens, represented by phage-fused peptides that mimic *Leishmania infantum* antigens, were selected according to their affinity to antibodies from asymptomatic and symptomatic VL dogs' sera.

**Methodology/Main Findings:**

Twenty phage clones were selected after three selection cycles, and were evaluated by means of *in vitro* assays of the immune stimulation of spleen cells derived from naive and chronically infected with *L. infantum* BALB/c mice. Clones that were able to induce specific Th1 immune response, represented by high levels of IFN-γ and low levels of IL-4 were selected, and based on their selectivity and specificity, two clones, namely B10 and C01, were further employed in the vaccination protocols. BALB/c mice vaccinated with clones plus saponin showed both a high and specific production of IFN-γ, IL-12, and GM-CSF after *in vitro* stimulation with individual clones or *L. infantum* extracts. Additionally, these animals, when compared to control groups (saline, saponin, wild-type phage plus saponin, or non-relevant phage clone plus saponin), showed significant reductions in the parasite burden in the liver, spleen, bone marrow, and paws' draining lymph nodes. Protection was associated with an IL-12-dependent production of IFN-γ, mainly by CD8^+^ T cells, against parasite proteins. These animals also presented decreased parasite-mediated IL-4 and IL-10 responses, and increased levels of parasite-specific IgG2a antibodies.

**Conclusions/Significance:**

This study describes two phage clones that mimic *L. infantum* antigens, which were directly used as immunogens in vaccines and presented Th1-type immune responses, and that significantly reduced the parasite burden. This is the first study that describes phage-displayed peptides as successful immunogens in vaccine formulations against VL.

## Introduction

Leishmaniasis is a disease with a wide spectrum of clinical manifestations caused by different species of protozoa belonging to the *Leishmania* genus [1]. The disease presents a high morbidity and mortality throughout the world, where 350 million people in 98 countries are at risk of contracting the infection. Moreover, approximately 1.0 to 1.5 million cases of cutaneous leishmaniasis (CL), and 200,000 to 500,000 cases of visceral leishmaniasis (VL), are registered annually [2], [3]. Canine visceral leishmaniasis (CVL) due to *Leishmania infantum* is a major global zoonosis that is potentially fatal to dogs [4]. This disease can be found in Southern Europe, Africa, Asia, and Central and South America; and is endemic in approximately 70 countries worldwide [5]. CVL is expanding its geographic distribution throughout the Western hemisphere, where it now occurs from northern Argentina to the United States [6], even reaching as far as provinces of southern Canada [7]. This disease is also an important concern in non-endemic countries, where imported sick or infected dogs constitute a veterinary and public health problem [6], [7].

The treatment of leishmaniasis is still based on the use of the parenteral administration of pentavalent antimonial compounds; however, several side effects reported by patients, and increased parasite resistance have produced serious problems [8], [9]. Therefore, the development of new strategies to prevent leishmaniasis has become a high priority [10].

The evidence of life-long immunity has inspired the development of vaccination protocols against the disease, but few have progressed beyond the experimental stage. Some studies have demonstrated that the Type-1 cells mediated immunity is important to a protective response against CL [11]–[15]. In addition, Th1 cells response has also been correlated with the protection against VL [16]. In this context, the protective immunity in murine VL primarily depends on an IL-12-driven Th1 cells response, leading to an IFN-γ and IL-2 production. Substantial up regulation of inducible NO synthase by IFN-γ generates NO from splenic and liver cells, thereby controlling parasite replication in these organs [16], [17]. By contrast, TGF-β, IL-10, and IL-13 represent major disease promoting cytokines, in turn leading to the suppression of the Th1 response [18]. Low levels of IL-4 can enhance vaccine-induced protection by indirectly increasing IFN-γ production by Th1 cells [19]. Thus, antigens that are capable of stimulating the development of a Th1 immune response in murine models, primed by the production of cytokines, such as IFN-γ and IL-12, in *in vitro* spleen cell cultures, could be considered promising vaccine candidates for use against *Leishmania* infection [20], [21].

One interesting approach toward the discovery of new antigens with biotechnological applications has been based on phage display technology [22]. This technique is based on DNA recombination, resulting in the expression of foreign peptide variants on the outer surface of phages. Using an *in vitro* selection process, based on binding affinity and so-called bio-panning cycles, peptides exposed in the selected phage clones are analyzed by DNA sequencing and identified [23]–[25]. Phage display has been used to identify mimotopes (peptides that mimic linear, discontinuous, and even non-peptide epitopes [26]) to be applied in the diagnoses of diseases, including malaria [27]–[29], leishmaniasis [30], toxoplasmosis [31], [32], and Chagaś disease [33]; as well as employed as vaccine candidates in the cysticercosis [34], trichinellosis [35], and Alzheimeŕs disease [36].

In the present study, phage display was used to identify mimotopes expressed on a foreign surface of phages, to be applied as vaccine candidates against *L. infantum*. Clones from a phage library were selected by antibodies present in sera samples of dogs with asymptomatic and symptomatic VL, and they were used for the *in vitro* immune stimulation of spleen cells obtained from naive or chronically infected with *L. infantum* BALB/c mice; in an attempt to select those able to stimulate a higher production of IFN-γ and lower levels of IL-4. Two phage clones presenting the best results of specificity and selectivity were selected and evaluated in vaccination experiments in BALB/c mice. Both clones, namely B10 and C01, were able to induce a Th1 immune response in the vaccinated mice, and were protective against *L. infantum* infection. In this context, these two antigens, identified by a non-described study using phage display and leishmaniasis; could be considered candidates to compose an effective vaccine against VL.

## Materials and Methods

### Ethics Statement

Experiments were performed in compliance with the National Guidelines, as set forth by the Institutional Animal Care (Law number 11.794, 2008), and the Committee on the Ethical Handling of Research Animals from the Federal University of Minas Gerais (UFMG), who approved this study under protocol number 043/2011. In addition, the owners of the domestic dogs (*Canis familiaris*) gave permission for their animals to be used in this study.

### Mice and parasites

Female BALB/c mice (8 weeks of age) were obtained from the breeding facilities of the Department of Biochemistry and Immunology, Institute of Biological Sciences (ICB), UFMG; and were maintained under specific pathogen-free conditions. Experiments were carried out using the *L. infantum* (MOM/BR/1970/BH46) strain. Parasites were grown at 24°C in Schneider's medium (Sigma-Aldrich, St. Louis, MO, USA), supplemented with 10% heat-inactivated fetal bovine serum (FBS; Sigma-Aldrich), 20 mM L-glutamine, 200 U/mL penicillin, and 100 µg/mL streptomycin, pH 7.4. The soluble *Leishmania* antigenic (SLA) extract was prepared from stationary promastigotes of *L. infantum*, after few passages in liquid culture, as described [11]. Briefly, 2×10^8^ promastigotes per mL, in a volume of 5 mL, were washed 3 times in 5 mL of cold sterile phosphate-buffered saline (PBS). After five cycles of freezing and thawing, the suspension was centrifuged at 8,000×*g* for 20 min at 4°C, and the supernatant containing SLA was collected in 500 µL aliquots and stored at −70°C, until use. The protein concentration was estimated by Bradford method [37].

### Sera samples

The sample size used in this study consisted of 60 domestic dogs, made up of males and females of different breeds and ages, collected from an endemic area of Belo Horizonte, Minas Gerais, Brazil. Sera of CVL were selected on the basis of two serological tests (IFAT [IFAT-LVC Bio-Manguinhos kit] and ELISA [EIE-LVC Bio-Manguinhos kit], both from Bio-Manguinhos, Fiocruz, Brazil) for *Leishmania spp*. Dogs with an IFAT titre <1/40 or ELISA reactivity below the cut-off value indicated by the manufacturer were considered to be seronegative. Animals with an IFAT titre> 1/40 and an ELISA value over the cut-off were considered to be seropositive. Thus, symptomatic dogs (n = 20) were those positive by IFAT and ELISA, but also with positive parasitological results by PCR-RFLP (restriction fragment length polymorphism), as well as presenting more than three clinical symptoms (weight loss, alopecia, adenopathy, onychogryposis, hepatomegaly, conjunctivitis; and exfoliative dermatitis on the nose, tail, and ear tips). Asymptomatic dogs (n = 20) presented positive serological (IFAT and ELISA) and parasitological (PCR-RFLP) results, but they did not present any clinical signals or symptoms of leishmaniasis. Healthy dogs (n = 20) were selected from an endemic area of Belo Horizonte, but they presented negative serological and parasitological results, as well as were free of any clinical signs or symptoms of leishmaniasis.

### Purification of antibodies by coupling in protein G microspheres

In order to obtain microspheres incorporated with IgG molecules derived from different será groups, a purification was performed using the coupling to magnetic microspheres (beads), conjugated to protein G (Dynabeads, Invitrogen). For this, 2×10^9^ microspheres were prepared by washing 3 times with 1 mL of 0.1 M MES buffer pH 5.0, been separately added to them: 375 µL of a pool of sera of healthy dogs (n = 20), 195 µL of a pool of sera of asymptomatic CVL (n = 20), or 300 µL of a pool of sera of symptomatic CVL (n = 20). After this, an incubation of 40 min was performed with each pool of sera and microspheres, under constant stirring and at room temperature. The microspheres coupled with each antibodieś group were then washed 3 times using 1 mL of 0.1 M MES buffer pH 5.0, in order to remove the non-adhered molecules. Next, the system was washed twice with 1 mL of 0.2 M triethanolamine buffer pH 8.2, and resuspended in 1 mL of covalent coupling buffer (containing 20 mM dimethy pimelimidate/HCl diluted in triethanolamine buffer) for 30 min, under constant stirring, at room temperature. The neutralization of unbound sites was made by incubating 1 mL of 50 mM Tris-base pH 7.5, for 15 min at room temperature. The system (beads plus antibodies) was washed 3 times with 1 mL TBS-T (50 mM Tris-HCl pH 7.5, 150 mM NaCl, and 0.1% Tween 20) buffer, blocked by addition of 2 mL of a blocking solution (5% BSA diluted in TBS-T) for 1 h at 37°C, and resuspended in 200 µL of TBS (50 mM Tris-HCl pH 7.5 and 150 mM NaCl) buffer. To verify the coupling of the generated beads-IgG systems (using healthy dogs, asymptomatic and symptomatic CVL sera), 5 µL of them were individually incubated with an anti-dog IgG peroxidase antibody (1∶5,000 dilution), for 1 h at 37°C. After this, they were washed 3 times with 1 mL of TBS-T, and the reaction was revealed by adding TMB substrate, being then stopped by adding 25 µL H_2_SO_4_ 2 N. The optical density was read in an ELISA microplate spectrophotometer (Molecular Devices, Spectra Max Plus, Canada), at 450 nm.

### Bio-panning cycles

To carry out a negative selection process in the bio-panning cycles, 1×10^11^ viral particles from a phage library containing random seven-peptides fused to a minor coat protein of M13 filamentous phages (Ph.D.-C7C library, New England BioLabs, USA) were diluted in 190 µL of TBS-T buffer. The mixture was incubated for 30 min at room temperature with the microspheres coupled to IgGs that had been purified from healthy dogs, and then precipitated by magnetic attraction to produce a Dynal Biotech support (12020). The supernatant containing the clones that were not adhered to the IgGs was recovered and transferred to a new tube, been this procedure repeated for three times. After this, a positive selection process was performed. For this, the supernatant containing the previously recovered phage clones were transferred to a new tube containing the microspheres coupled to IgGs purified from asymptomatic CVL, and incubation occurred for 30 min at room temperature. After, the supernatant was removed, and the remained bound phages to the IgGs were washed 10 times with 1 mL of TBS-T, and eluted in 500 µL of 0.2 M glycine buffer, pH 2.0. Next, 75 µL of 1 M Tris-base pH 9.0 was added to neutralize the acid pH. Subsequently, the recovered phage clones were transferred to a new tube containing the IgGs purified from asymptomatic CVL, and the process was repeated for three times. After this, the recovered phage clones were transferred to a new tube containing the IgGs that had been purified from symptomatic CVL, and the process was also repeated for three times, when the selected clones reactive to IgGs from asymptomatic and symptomatic sera of CVL were then recovered and titrated.

### Titration of phage clones

Phage clones were diluted 10^−1^ to 10^−11^ in 500 µL of sterile TBS buffer, mixed with an *Escherichia coli* culture (OD_600nm_ ∼ 0.5), and plated on LB agar plates containing 1 mL of a IPTG/X-gal solution (composed by 200 mg/mL isopropyl β-D-1-thiogalactopyranoside and 20 mg/mL 5-bromo-4-chloro-3-indolyl-β-D-galactoside, diluted in dimethyl sulfoxide PA). Colonies were individually quantified, and the titration was performed for each bio-panning cycle. After the 3^rd^ round of positive selection using IgGs from CVL sera, 96 colonies selected from the plate were added in 200 µL of LB (Luria Bertani) medium in a sterile culture plate (BD Falcon TM clear, 96-well microtest TM plate), then the plate was sealed and incubated for 5 h, under constant stirring, at 37°C. After incubation, the plate was centrifuged for 20 min by 2,250×*g*, and the supernatant was transferred to a new plate, where a PEG/NaCl solution (20% PEG 8,000 and 2.5 M NaCl) was added (1/6 of the total volume of supernatant), and the plate was incubated for 16 h at 4°C. After, the plate was centrifuged for 1 h, the supernatant was removed, and the pellet was resuspended in 500 µL of a solution composed by 10 mM Tris-HCl pH 8.0, 1 mM EDTA, and 4 M NaI. The plate was shaken vigorously for 5 min, when 250 µL of a 70% ethanol solution was added. After having been incubated for 10 min, the plate was centrifuged (2,250×*g* at 4°C for 10 min), and the supernatant was discarded. The pellet containing the DNA of interest of each clone was washed with 500 µL of 70% ethanol, and again centrifuged. Finally, the DNA was diluted in 20 µL of ultra-pure water, and its quality was evaluated in a 1% agarose gel, which was stained with an ethidium bromide solution (10 µg mL^−1^). The individual DNA of clones was used for the sequencing and identification of target peptides.

### DNA sequencing and bioinformatics

The sequencing reaction was performed using 500 ng of DNA for each selected phage clone, 5 pmol primer 96 gIII (5′-OH CC TCA TAG TTA GCG TAA CG-3′, Biolabs), plus a pre-mix (Dye Terminator Cycle ET Journal Kit, Amersham Biosciences). Thirty-five cycles were performed in a thermocycler, under the following conditions: denaturation at 95°C for 20 sec, ringing at 58°C for 15 sec, and extension at 60°C for 60 sec. Ten microliters of the generated amplicons were precipitated with 1 µL of ammonium acetate (1∶10 ratio), added with 27.5 µL of ethanol PA. The plate was centrifuged for 45 min to 2,432×*g*, when the supernatant was discarded and 150 µL of 70% ethanol was added to the pellet. The resuspended DNA was centrifuged for 10 min at 2,432×*g*, and the supernatant was discarded again. The plate was inverted on a paper towel and, in this position, was centrifuged at 486×*g* for 1 min. Next, the plate was covered for 5 min, until the complete evaporation of the remaining ethanol had been achieved. The pellet was resuspended in dilution buffer, and the sequencing was performed in a MegaBace 1000 automatic sequencer (Amersham Biosciences). Peptide sequences of 20 valid phage clones were deduced using the Expasy server (www.expasy.org), and analyzed with the Pepbank [38], MimoDB [39] and SAROTUP [40] programs to identify possible false-positive sequences.

### Evaluation of the *in vitro* immune stimulation

BALB/c mice (n = 8) were subcutaneously infected with 1×10^7^ stationary phase promastigotes of *L. infantum*, and they were monitored by 10 weeks. Liver, spleen, paws' draining lymph nodes (dLN), and bone marrow (BM) of the animals were collected for parasite quantification, following a limiting-dilution technique [11]. The parasite load was performed to confirm that the animals were chronically infected (data not shown). After this, spleen cells were collected, pooled and *in vitro* cultured in 24-well plates (Nunc, Nunclon^®^, Roskilde, Denmark), at 5×10^6^ cells per mL, in duplicate. Cells were incubated in RPMI 1640 medium (Sigma-Aldrich; non-stimulated control), or separately stimulated with each phage clone (20 individual clones, with 1×10^10^ phages per well), at 37°C in 5% CO_2_ for 48 h. The same experimental conditions were performed using spleen cells of naive mice. Wild-type and random non-specific phage clones were used like controls. The IFN-γ and IL-4 levels were determined in the culture supernatants, using commercial kits (BD OptEIA, Pharmingen, San Diego, CA, USA), according to manufactureŕ instructions. Experiments were repeated twice and presented similar results.

### Immunization and challenge infection

After the *in vitro* immunogenicity experiments, two from 20 phage clones were selected, namely B10 and C01, based on their polarized Th1 immune response, and were used in the *in vivo* experiments. For this, BALB/c mice (n = 8, per group) were vaccinated subcutaneously in their left hind footpad with two selected clones or wild-type phage (1×10^11^ phages, each one), associated with 25 µg of saponin (*Quillaja saponaria* bark saponin; Sigma Aldrich), only adjuvant or received the diluent (saline). Three doses were administered at 2-week intervals. Four weeks after the last immunization, animals (n = 4, per group) were euthanized for the analysis of the immune response elicited by vaccination. At the same time, the remaining animals were infected subcutaneously in their right hind footpad with 1×10^7^ stationary promastigotes of *L. infantum*, and they were follow-up for 10 weeks. Experiments were repeated twice and presented similar results.

### Estimation of parasite load

The liver, spleen, BM and dLN were collected of the euthanized animals for parasite quantification, using a limiting-dilution protocol [11]. Briefly, organs were weighed and homogenized using a glass tissue grinder in sterile PBS. Tissue debris were removed by centrifugation at 150 × *g*, and cells were concentrated by centrifugation at 2,000 × *g*. Pellets were resuspended in 1 mL of Schneider's insect medium supplemented with 20% FBS. Two hundred and twenty microliters were plated onto 96-well flat-bottom microtiter plates (Nunc), and diluted in log-fold serial dilutions using supplemented Schneider's medium, to a 10^−1^ to 10^−12^ dilution. Each sample was plated in triplicate and read 7 days after the beginning of the culture, at 24°C. Pipette tips were discarded after each dilution to avoid carrying adhered parasites from one well to another. Results are expressed as the negative log of the titer (*i.e*., the dilution corresponding to the last positive well) adjusted per microgram of tissue.

### Cytokine production

Splenocytes cultures and cytokine assays were performed before infection and at 10^th^ week after challenge, as described [11]. Briefly, single-cell preparations from spleen tissue were plated in duplicate in 24-well plates (Nunc), at 5×10^6^ cells per mL. Cells were incubated in RPMI 1640 medium (background control), or separately stimulated with SLA (20 µg mL^−1^), or individual phage clones (1×10^10^ phages, each one), at 37°C in 5% CO_2_ for 48 h. The IFN-γ, IL-4, IL-10, IL-12, and GM-CSF levels were determined in the culture supernatants, using commercial kits (Pharmingen), according to manufactureŕ instructions. In order to block IL-12, CD4, and CD8 mediated T cells cytokine release, spleen cells of mice vaccinated and infected were *in vitro* stimulated with SLA (20 µg mL^−1^), and incubated in the presence of 5 µg mL^−1^ of monoclonal antibodies (mAb) against mouse IL-12 (C017.8), CD4 (GK 1.5), or mouse CD8 (53–6.7). Appropriate isotype-matched controls – rat IgG2a (R35-95) and rat IgG2b (95-1) – were employed in the assays. Antibodies (no azide/low endotoxin) were purchased from BD (Pharmingen).

### Analysis of the humoral response

SLA-specific IgG1 and IgG2a antibodies were measured by ELISA, as described elsewhere [11]. Briefly, previous titration curves were performed to determine the most appropriate antigen concentration and antibody dilution to be used. Falcon flexible microtiter immunoassay plates (Becton Dickinson) were coated with SLA (1.0 µg per well) in 100 µL coating buffer (50 mM carbonate buffer) pH 9.6 for 18 h, at 4°C. After this, free binding sites were blocked using 200 µL of TBS-T buffer containing 5% casein for 1 h, at 37°C. After washing the plates 7 times using TBS-T, they were incubated with 100 µL of individual sera for 1 h at 37°C. Samples were diluted 1∶100 in TBS-T containing 0.5% casein solution. Plates were washed 7 times using TBS-T, and incubated with the peroxidase-labeled antibodies specific to mouse IgG1 or IgG2a isotypes (Sigma Aldrich) diluted at 1∶5,000, and incubated for 1 h at 37°C. After washing 7 times with TBS-T, the reaction was developed through incubation with H_2_O_2_, *orto-*phenylenediamine and citrate-phosphate buffer pH 5.0, for 30 min in the dark. The reaction was stopped by adding 25 µL H_2_SO_4_ 2 N, and optical density was read in an ELISA microplate spectrophotometer, at 492 nm.

### Statistical analysis

The statistical analysis was made using the GraphPad Prism software (version 6.0 for Windows). Results were expressed by mean ± standard deviation (SD) of the groups. Outliers were evaluated using ROUT test, and excluded from statistical analyses. The normality analysis of the data was performed using the D'Agostino & Pearson test. Statistical analysis of the results of immunized and/or infected mice was performed by one-way analysis of variance (ANOVA), using Tukey's post-test for multiple comparisons between the groups. Differences were considered significant with *P* <0.05. Data showed in this study are representative of two independent experiments, which presented similar results.

## Results

### Selection of the phage clones

**Figure 1:**
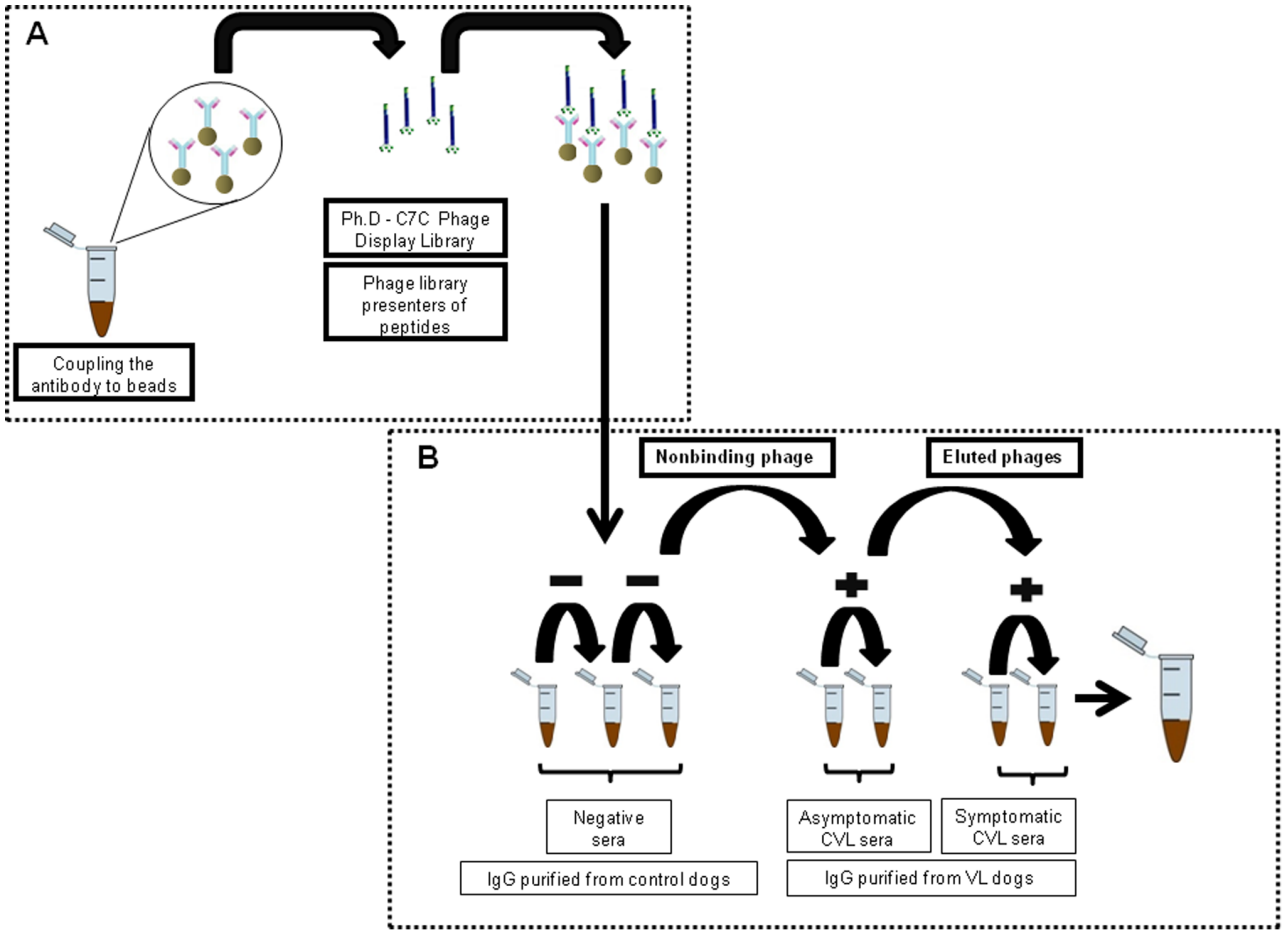
Schedule of technical protocol used in the bio-panning cycles of phage display.

First, the phage clones selection was performed by excluding those that were recognized by antibodies present in sera samples of healthy dogs. After, a positive selection process was performed, by recovering the phage clones that were recognized by antibodies present in the sera samples of dogs with asymptomatic and symptomatic VL. The technical protocol is summarized in the Figure 1. Approximately 96 clones were randomly selected from individual colonies, and their DNA sequences were amplified by PCR and sequenced. Twenty clones had their sequences clearly identified (Table 1), and an alignment showed that no identical consensus motif could be detected between them (data not shown). In addition, none of the 20 sequences selected were identified as previously described sequences, or as non-specific binders to the commons reagents used in the bio-panning cycles (data not shown).

**Table 1:** Amino acid sequences from target peptides present in the selected phage clones.

Clone	Sequence
A10	LLSSKTL
B10	LSFPFPG
C01	FTSFSPY
C02	QATHFHS
D09	LASLPFR
E03	THVFSWI
E10	ACDPSPN
E11	TPSLHRS
F01	TAMARSA
F03	VALLPHH
F04	QSPPALL
F08	FSLLGSL
F09	VLLGPFP
F11	YPFSLLH
G02	SLGPQIK
G05	MSPTYLL
G09	DRAALSL
G11	ALTPQLL
G12	QTSPPLA
H05	FPLFGLS

**Figure 2:**
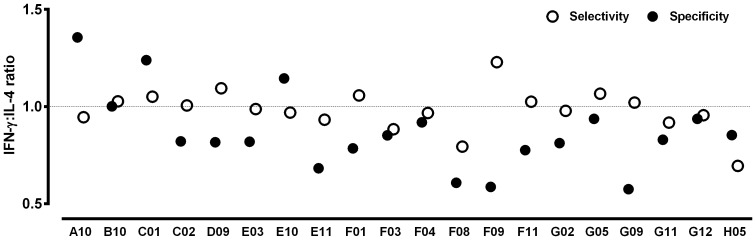
Evaluation of the selectivity and specificity of the selected phage clones. Spleen cells suspensions were obtained from naive or chronically infected with *L. infantum* BALB/c mice. Cells were non-stimulated (medium; background control), or separately stimulated with each phage clone, as well as the wild-type and non-relevant clones (1×10^10^ phages, each one) for 48 h at 37°C, 5% CO_2_. IFN-γ and IL-4 levels were measured in culture supernatants by capture ELISA. The black circles indicate the specificity of the phage clones, which was calculated by dividing the IFN-γ and IL-4 values obtained of each evaluated individual clone by respective values of these cytokines obtained after the wild-type phage stimulus, using spleen cells derived from naive mice. With the corrected values, the ratio between IFN-γ and IL-4 with these new values was calculated, and the specificity of the each clone was defined. The white circles indicate the selectivity, which was calculated by dividing the IFN-γ and IL-4 values obtained of each evaluated individual clone by respective values of these cytokines obtained after the non-relevant phage stimulus, using spleen cells derived from infected mice. With the corrected values, a ratio between IFN-γ and IL-4 using these new values was calculated, and the selectivity of each clone was defined.

Spleen cells derived from naive or chronically infected with *L. infantum* BALB/c mice were collected, pooled and separately stimulated with each one of the 20 phage clones, at which time the IFN-γ and IL-4 levels were determined in the cultures supernatants. A wild-type phage clone, identical to the phage clones present in the library, but that did not express foreign exogenous mimotopes; a random non-specific to *Leishmania* phage clone (NMSDFLRIQLRS), and non-stimulated cells (medium, background) were used as controls (Supplementary Figure). To select the phage clones that presented the best results of immune stimulation, their specificity and selectivity were calculated, comparing the results obtained after the stimulation of spleen cells with the values obtained from wild-type and non-relevant phages. The specificity was defined as the ability of a clone to bind to its target, based on the presence of mimotopes displayed on its surface; whereas the selectivity was defined as the ability of a clone to identify its cognate target in a mixture of different targets [41]. Initially, the specificity was calculated by dividing the IFN-γ and IL-4 values obtained of each evaluated individual clone by respective values of these cytokines obtained after the wild-type phage stimulus, using spleen cells derived from naive mice. Then, with the corrected values, a ratio between IFN-γ and IL-4 using these new values was calculated, and the generated number was defined as the specificity of clone. In the same way, the selectivity was calculated by dividing the IFN-γ and IL-4 values obtained of each evaluated individual clone by respective values of these cytokines obtained after the non-relevant phage stimulus, using spleen cells derived from infected mice. Then, with the corrected values, a ratio between IFN-γ and IL-4 using these new values was calculated, and the generated number was defined as the selectivity of clone (Fig. 2). The phage clones that presented a ratio ≥ 1.0 were selected, being this case observed with the B10 and C01 phages; in this context, these molecules were evaluated as vaccine candidates against *L. infantum* infection.

### Immunogenicity of the selected phage clones

**Figure 3:**
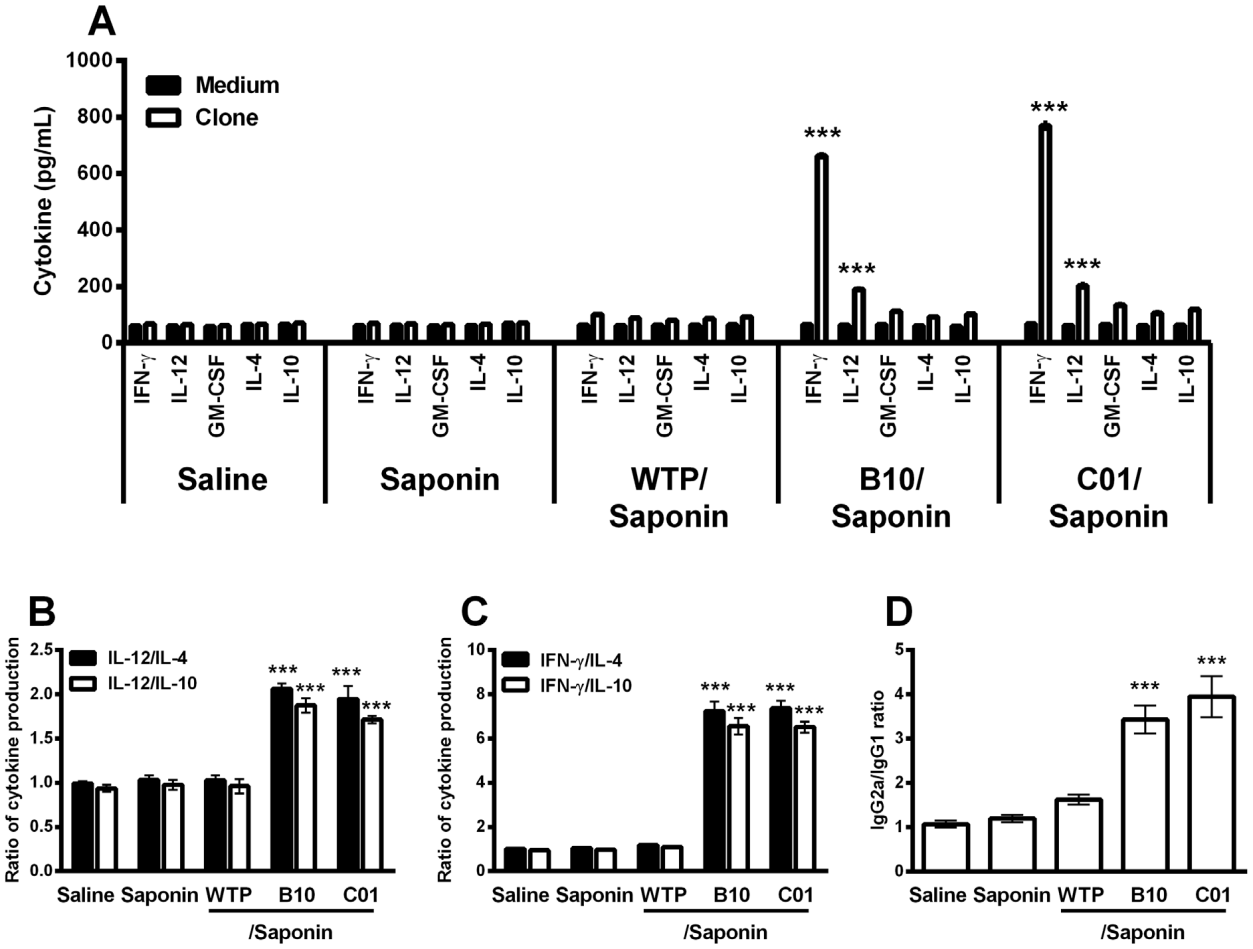
Cellular and humoral response induced in BALB/c mice by immunization with B10 or C01 phage clones plus saponin. Single cells suspensions were obtained from the spleen of mice, four weeks after the last immunization. Cells were non-stimulated (medium; background control), or separately stimulated with individual clones (1×10^11^ phages per mL, each one) for 48 h at 37°C, 5% CO_2_. IFN-γ, IL-12, GM-CSF, IL-4, and IL-10 levels were measured in culture supernatants by capture ELISA (**A**). Each bar represents the mean ± standard deviation (SD) of the groups. Statistically significant differences in the IFN-γ and IL-12 levels between the B10 or C01 clone plus saponin groups and control mice (saline, saponin and wild-type phage plus saponin groups) were observed (***P <0.0001). The ratio between IL-12/IL-10 and IL-12/IL-4 levels (**B**), and between IFN-γ/IL-10 and IFN-γ/IL-4 levels (**C**) are also showed. Statistically significant differences in the ratios between the B10 or C01 clone plus saponin groups and control groups were also observed (***P <0.0001). The ratio between the *Leishmania*-specific IgG1 and IgG2a antibodies was obtained from the different groups, and statistically significant differences between the B10 or C01 clone plus saponin and control groups were observed (***P <0.0001) (**D**).

The immunogenicity of B10 and C01 clones was evaluated in BALB/c mice, four weeks after the last vaccine dose (Fig. 3). After having undergone *in vitro* immune stimulation, spleen cells from vaccinated mice significantly produced high levels of IFN-γ and IL-12 than did those secreted by spleen cells from all control groups. No significant production of IL-4 and IL-10 could be observed in any experimental groups after stimulation with individual clones (Fig. 3A). The ratios between IL-12/IL-4 and IL-12/IL-10 (Fig. 3B), and between IFN-γ/IL-4 and IFN-γ/IL-10 (Fig. 3C) showed that vaccinated animals with B10/saponin or C01/saponin presented an elevated Th1 immune response after receiving a specific phage-stimulus. In addition, mice vaccinated presented a humoral response with a higher predominance of SLA-specific IgG2a isotype, in comparison to the IgG1 levels (Fig. 3D).

### Protective efficacy of the phage clones against *L. infantum*

**Figure 4:**
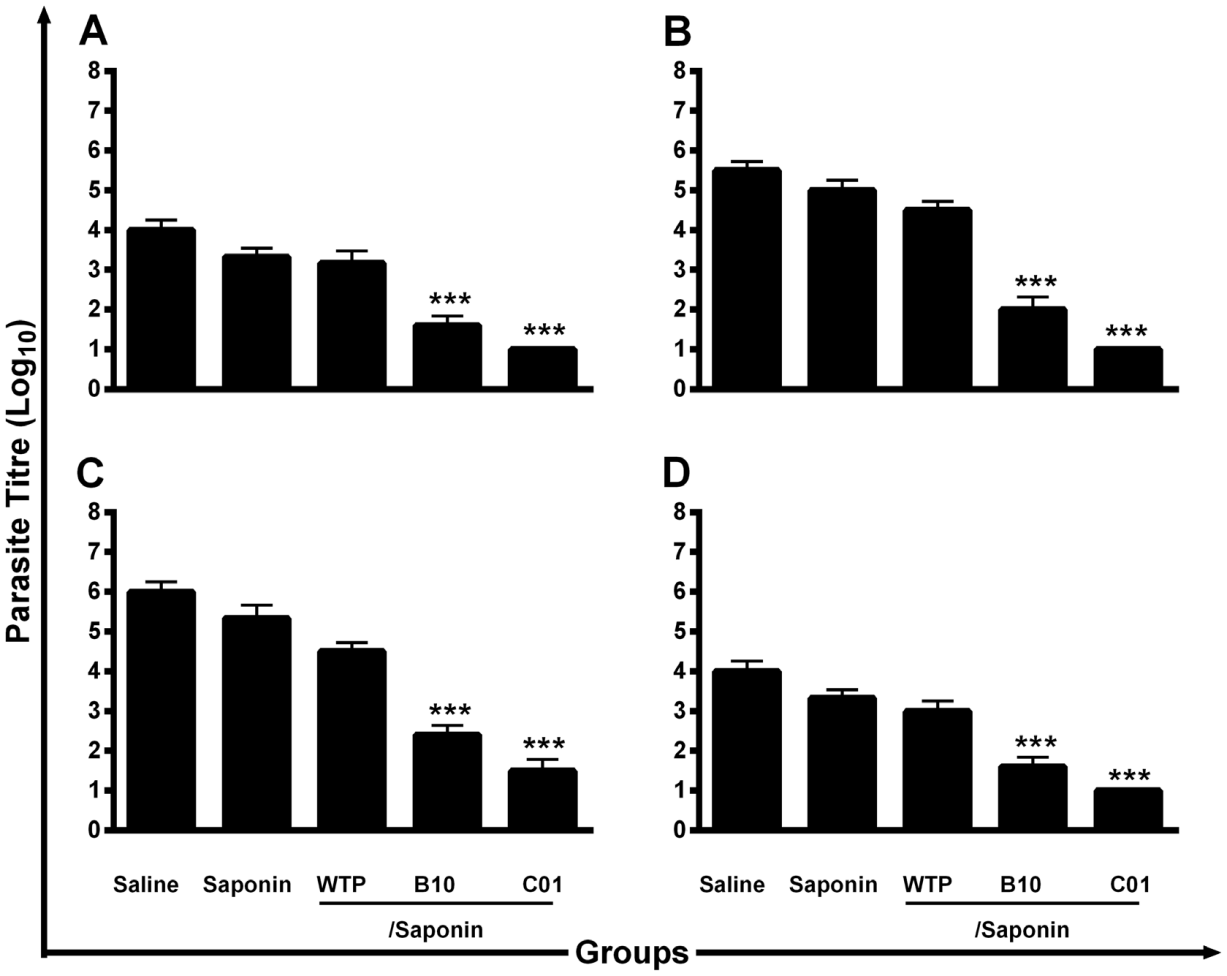
Protection of BALB/c mice vaccinated with phage clones plus saponin against *L. infantum.* Mice inoculated with saline, saponin, wild-type phage (WTP), B10 or C01 phage clones plus saponin were subcutaneously infected with 1×10^7^ stationary phase promastigotes of *L. infantum.* The number of parasites in the liver (**A**), spleen (**B**), pawś draining lymph nodes (**C**), and bone marrow (**D**) was measured, 10 weeks after challenge by a limiting-dilution technique. Each bar represents the mean ± standard deviation (SD) of the groups. Statistically significant differences in the parasite load in all evaluated organs between the B10 and C01 clones plus saponin and control groups are showed (***P <0.0001). Data shown are representative of two independent experiments, which presented similar results.

Next, this study analyzed whether the immunization with the B10 or C01 clones plus saponin was in fact able to induce protection against *L. infantum*. After the three vaccine doses, the infection was performed and the animals were followed-up over a 10-weeks period, at which time the parasite burden in the liver, spleen, dLN, and BM was determined. Significant reductions in the number of parasites were observed in all of the evaluated organs of the vaccinated mice, as compared to those that received saline, or that were immunized with the wild-type phage clone plus saponin (Fig. 4). Vaccinated mice with B10 or C01 clones plus saponin, as compared to the saline group, as an infection control; presented significant reductions in the parasite load in the liver (2.5- and 5.3-log reductions, respectively; Fig. 4A), spleen (2.8- and 4.4-log reductions, respectively; Fig. 4B), dLN (2.5- and 4.0-log reductions; respectively, Fig. 4C), and BM (2.5- and 5.3-log reductions, respectively; Fig. 4D). In the same way, immunized mice with B10 or C01 clones plus saponin, as compared to the wild-type phage clone plus saponin group, as a phage control; showed significant reductions in the parasite load in the liver (2.0- and 4.2-log reductions, respectively), spleen (2.3- and 3.6-log reductions, respectively), dLN (1.9- and 3.0-log reductions; respectively), and BM (1.9- and 4.0-log reductions, respectively). In both cases, the results showed an effective protection induced by B10 and C01 phage clones against the *Leishmania* infection, which could be represented by exposed mimotopes in their foreign surface, mainly leading in count by their absence in the wild-type phage clone.

### Cellular response elicited after L. infantum infection

**Figure 5:**
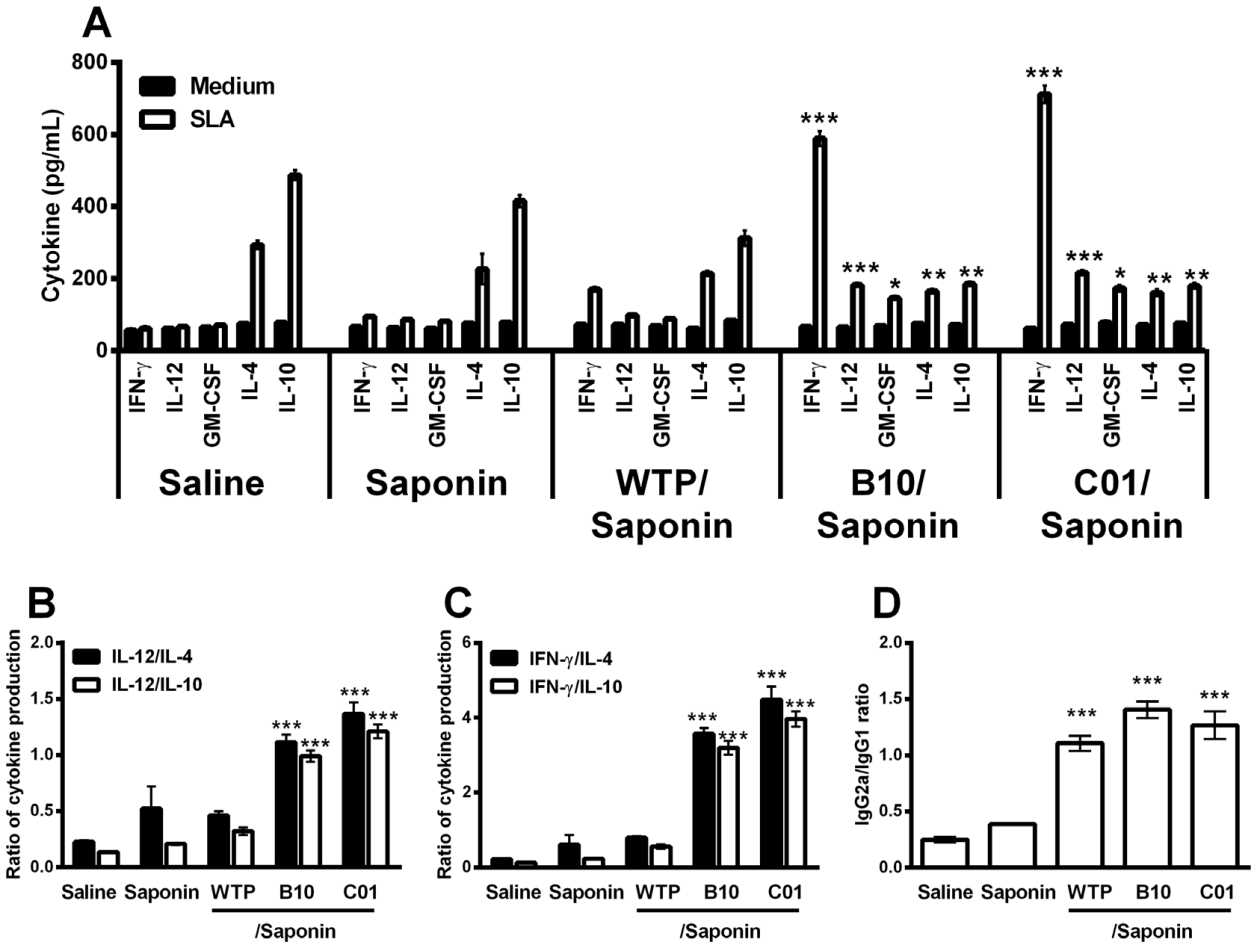
Analysis of the cellular and humoral response in BALB/c mice after *L. infantum* challenge infection. BALB/c mice (n = 8, per group) received saline or were immunized subcutaneously in their left hind footpad with 25 µg of saponin, and with B10, C01 or wild-type phage clones (1×10^11^ phages, each one), associated with saponin. Three doses were administered at 2-week intervals, and four weeks after the last immunization, the animals (n = 4) were infected subcutaneously in their right hind footpad with 1×10^7^ stationary promastigotes of *L. infantum*, and were followed for 10 weeks. Single-cells suspensions were obtained from the spleens of mice in this time, and were non-stimulated (medium; background control), or stimulated with SLA (20 µg mL^−1^) for 48 h at 37°C, 5% CO_2_. Levels of IFN-γ, IL-12, GM-CSF, IL-4 and IL-10 were measured in culture supernatants by capture ELISA. Each bar represents the mean ± standard deviation (SD) of the cytokines levels of the different groups (**A**). Statistically significant differences between the B10 and C01 clones plus saponin and control groups (saline, saponin and wild-type phage plus saponin groups) were observed (***P <0.0001). The ratio between IL-12/IL-10 and IL-12/IL-4 levels (**B**), and between IFN-γ/IL-10 and IFN-γ/IL-4 levels (**C**), are also showed. Statistically significant differences between the B10 and C01 clones plus saponin and control groups were observed (***P <0.0001). The ratio between SLA-specific IgG1 and IgG2a antibodies levels were calculated and statistically significant differences between the B10 and C01 clones groups and the saline and saponin were also observed (*P <0.005) (**D**).

**Figure 6:**
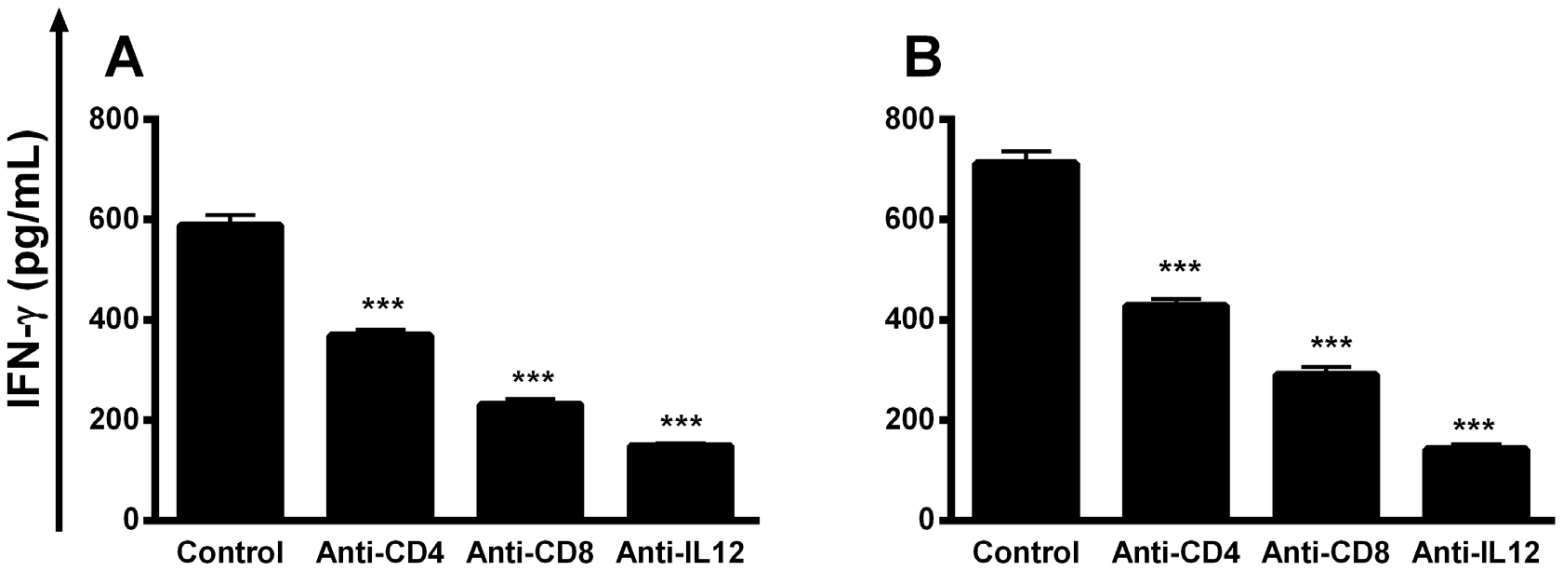
Involvement of IL-12, CD4^+^ and CD8^+^ T cells in the IFN-γ production after *L. infantum* infection. Single-cells suspensions were obtained from the spleens of mice that were immunized with B10 clone plus saponin (**A**) or C01 clone plus saponin (**B**), 10 weeks after *L. infantum* infection. Levels of IFN-γ were measured in culture supernatants by capture ELISA of spleen cells cultures stimulated with SLA (20 µg mL^−1^) for 48 h at 37°C, 5% CO_2_. Cultures were incubated in the absence (positive control) or in the presence of 5 µg mL^−1^ of monoclonal antibodies (mAb) against mouse IL-12 (C017.8), CD4 (GK 1.5), or mouse CD8 (53-6.7). Statistically significant differences between non-treated control cells and cultures incubated with anti-CD4, anti-CD-8 and anti-IL-12 monoclonal antibodies were observed (***P <0.0001). Each bar represents the mean ± standard deviation (SD) of the IFN-γ levels of the different groups.

The production of cytokines in the supernatants of spleen cell cultures stimulated with SLA, 10 weeks after infection, was analyzed to evaluate the immunological correlates of protection induced by previous immunization. The spleen cells derived from mice vaccinated with B10 or C01 clones plus saponin produced higher levels of SLA-specific IFN-γ, IL-12, and GM-CSF, than did those secreted by spleen cells from control groups (Fig. 5A). In contrast, the SLA-driven production of IL-4 and IL-10 showed that vaccination with both phage clones induced no production of these cytokines in the vaccinated and infected animals. In this context, the ratios between IL-12/IL-4 and IL-12/IL-10 (Fig. 5B), and between IFN-γ/IL-4 and IFN-γ/IL-10 (Fig. 5C), indicated that vaccinated mice developed a Th1 response, which was maintained after infection. It was also possible to observe that vaccinated and infected mice presented a predominance of SLA-specific IgG2a antibodies, which was significantly higher than the IgG1 levels (Fig. 5D). The contribution of CD4^+^ and CD8^+^ T cells, as well as the dependence of IL-12 production for the SLA-specific IFN-γ response from the spleen cells of mice immunized with B10 or C01 phage clones plus saponin and infected with *L. infantum*, was also evaluated (Fig. 6). The IFN-γ production was significantly suppressed using an anti-CD8^+^ monoclonal antibody in the spleen cell cultures. The addition of anti-CD4^+^ or anti-IL-12 antibodies to the cultures also decreased the production of this cytokine, as compared to the control cells culture without treatment; however, the production of this cytokine proved to be greater than that produced by the use of the anti-CD8^+^ monoclonal antibody.

## Discussion

In a recent study, our group developed a subtractive phage display strategy that led to the identification of novel phage antigens, represented by specific mimotopes, which were applied to a sensitive and specific serodiagnosis of CVL [30]. In the present study, phage display was applied to the identification of antigens based on phage clones that could be evaluated in murine models, such as vaccines against VL. The selected phages expressing the target mimotopes were selected after bio-panning cycles, which had been performed using a negative selection process, employing sera samples from healthy dogs, as well as by a positive selection process, based on the use of sera samples from dogs with asymptomatic and symptomatic VL.

It has been proposed that parasite antigens that are recognizable by antibodies present in sera of dogs with asymptomatic VL may be well-associated with a protective response, and could represent potential vaccine candidates against leishmaniasis [42]. Here, the selected phage clones were used for *in vitro* immune stimulation experiments using spleen cells derived from naive or chronically infected with *L. infantum* BALB/c mice. Two clones were selected based on their ability of inducing a specific and selective higher production of IFN-γ, which was associated with low levels of IL-4. These clones were then evaluated in BALB/c mice, according to their ability to induce protection against *L. infantum* infection.

In the present study, spleen cells derived from naive or chronically infected BALB/c mice were used in the selection of the phage clones. Murine models have been used widely for the development of vaccines against VL [20], [43]. Studies employing the mouse model have led to the characterization of the immune mechanisms needed to develop organ specific immune responses, which would clear the parasites from the liver, but not from the spleen [43]. BALB/c mice infected with *L. donovani* or *L. chagasi/L. infantum* are the most widely studied models of VL, but they are also considered to be rather vulnerable, given that the infection progresses during the first two weeks and become chronic. In the murine VL, C57BL/6 mice mount an early Th1 immune response, which prevents the further growth of the parasite, in turn producing a self-healing phenotype [44], whereas susceptible BALB/c mice mount early Th2 response, which results in a non-healing lesion and an exaggeration of the disease [45]. In addition, the respective resistance and susceptibility of C57BL/6 and BALB/c strains depend not only on the Th1 and Th2 types of immune responses from CD4^+^ T cells, but also on the genetic background of the host [46]. In this manner, the immune response, following the infection of inbred mouse strains with viscerotropic *Leishmania* species, such as *L. donovani* and *L. infantum*; can be considered to be similar to those observed in the *L. major* mouse model. In this context, in the present study, spleen cells derived from naive and chronically infected with a high inoculums of *L. infantum* BALB/c mice were used to the *in vitro* immune stimulation experiments, in order to select only the candidate antigens able to subvert the undesirable Th2 profile in a mouse strain highly susceptible to *L. infantum* infection. Additional studies may well be carried out in order to extend the observations present herein of the protective efficacy of the B10 and C01 phage clones plus saponin vaccination to other mice models and/or experimental conditions.

Phage display, since its first description [47] and introduction into laboratory practice [48], has proven to be useful in selecting antigens derived from phages, either using the phage clone itself or the synthetic mimotopes, for vaccine development against diseases, such as Burkitt's lymphoma [49], melanoma [50], colorectal cancer [51], hepatitis B virus [52], and rotavirus [53]; or against pathogens, such as *Vibrio cholera* [54], *Candida albicans* [55], and *Brugia malayi* [56].

One could speculate that a major requirement for a successful vaccine will be based not only on the most effective way of inducing immunity, but also on the technical feasibility of its production [57]. Phage-displayed peptides as candidates possess two main advantages: first, peptides exposed in filamentous phages are antigenic and immunogenic. As very potent carriers, phages can be taken up and processed efficiently by mechanism of major histocompatibility complex class I and II pathways [58], [59]. Second, the amplification of phage is much simpler and inexpensive than the conventional route of peptide synthesis or production of recombinant proteins, besides of the final product is composed by a high number of virion copies, reflecting in a high exposition of the mimotopes to the immune system of the immunized hosts. Also, phages can replicate inside the phagocityc cells, and taken more time to be eliminated of the organism, in comparison to use of synthetic peptides or recombinant proteins, as well as they are not pathogenic to humans [60], [61]. Other important difference is based on that the immune stimulatory non-methylated CpG motifs, present in the phagé genome, can contribute to the activation of the immune system of the mammal hosts, through Toll-like receptors [62], [63], reducing the necessity of employ of immune adjuvants that are necessary to be incorporated together to synthetic peptides and recombinant proteins.

On the other hand, some factors could be considered to improve the protection observed in the present study using the B10 and C01 phage clones. In this context, these phages were screened from a Ph.D.-C7C Phage Display Peptide Library Kit, which displayed peptides fused to the pIII protein of them. Minor coat protein pIII is presented at five copies per virion, of which all five can be fused to short peptides without interfering in the phage infectivity. In contrast, major coat protein pVIII is presented at 2,700 copies per virion, of which 10% can be reliably fused to peptides. As a result, peptides expressed as pIII fusions are present at lower valency, whereas pVIII fusions are present at higher valency [61]. Therefore, protection observed in this study could be improved performing a construction of hybrid phage fusions of B10 and C01 epitopes to the pVIII of the filamentous phages. In addition, the incorporation of other protective epitopes could be considered an efficient strategy to develop a more protective phage-displayed multi-epitope based vaccine against *Leishmania* infection.

Usually, in studies using the phage display technology applied to select vaccine [64]–[66], or diagnosis [30], [67] candidates to diseases; the employed controls in the *in vivo* experiments are the own phage molecules that not express the foreign peptides, namely as wild-type phage. Normally, this control is derived of phage display library used in the selection process. In the present study, the wild-type phage used as a control was a filamentous phage derived from Ph.D.-C7C Phage Display Peptide Library Kit, which did not displayed exogenous peptides fused to the pIII protein, unlike to the other clones employed in the selection. This fact could be considered relevant, once the own phagé particles present proteins that can interact with the immune system of the hosts, leading to the development of an immune response specific to them, and interfering in the immune response specific to the target, in this case, to the selected phage-displayed mimotopes. In consequence, one could speculate that the higher or lower obtained protection in the vaccination experiments could have been influenced by molecules or parts of own phage, and did not due to the foreign mimotopes selected in the bio-panning cycles.

In the murine VL, the production of IL-4 or IL-10 mediated immune response inhibits the protective effects of the IFN-γ response, which may well be related to the deactivation of macrophages and the onset of the disease in the infected animals [68]. In studies evaluating vaccine candidates in the murine VL, the immunogens are usually administered in mice associated with Th1-type adjuvants and, after only a few weeks, they are infected and followed-up for a couple of months. In this time, spleen cells are collected and cultured *in vitro* with the antigens used in the immunization process, and/or with *Leishmania* extracts, in order to evaluate their immunogenicity. In this point, cytokines, such as IFN-γ and IL-12, markers of a Th1 response; and IL-4 and IL-10, indicators of a Th2 response, have their levels determined and, together with the results of the parasite burden, the efficacy of immunogens is evaluated [10], [15]. Thus, antigens capable of stimulating the development of a Th1 response, as represented by production of high levels of IFN-γ in mice naive or chronically infected with *Leishmania*, as opposite conditions, could be considered promising candidates for use as a vaccine against leishmaniasis.

Although the induction of a life-long protection against reinfection, after recovering from a primary challenge, demonstrates that a vaccine can be achieved, an effective candidate did not exist. Some antigens, such as GP63, GP46, LACK, CPB, CPA, A2, PSA-2, HASPB1, LeIF, and LCR1, have been tested like recombinant proteins, synthetic peptides, and/or DNA vaccines against leishmaniasis, but they have induced only partial protection [69]–[71]. Recently, to solve this problem, the use of remodeled live vaccines has become attractive, presenting notable arguments concerning immunogenicity and efficacy [69], [72], [73]. The main disadvantage of such vaccines may well be based on the difficulty of their large-scale production, as well as due to their difficult standardization [70].

The present study described, for the first time, the employ of the phage display technology to select antigens to be used as vaccines against *L. infantum*, in a known murine model. The immunization using B10 or C01 phage clones associated with saponin was able to induce a specific Th1 response in vaccinated BALB/c mice, which was characterized by an *in vitro* phages-specific production of IFN-γ, IL-12, and GM-CSF, combined with the presence of low levels of IL-4 and IL-10; as well as a predominance of SLA-specific IgG2a antibodies. After infection, immunized mice, as compared to the controls, including phage (wild-type phage) and mimotope (non-relevant phage) controls, displayed significant reductions in the parasite burden in all evaluated organs, which was correlated with a higher production of IFN-γ by their spleen cells; one of the main cytokines implicated in the protective immunity against *Leishmania* [28], [29], [31].

As regards the profile of T cells involved on this production, CD8^+^ T cells proved to be the major source of IFN-γ in the protected mice, since the depletion of these cells in the cultures of spleen cells stimulated with SLA, significantly nullified this response. Although previous reports have shown that the activation of both CD4^+^ and CD8^+^ cells subsets may be important for the killing of parasites in vaccinated mice using several parasite recombinant antigens [15], [32], [33], the present study's data suggest that CD4^+^ cells may contribute to a lesser extent to the induction of an IFN-γ mediated response elicited by immunization using B10 and C01 clones; being that the CD8^+^ T cells were the main resource of IFN-γ, and these cells could directly contribute to infection control through their direct cytotoxic effect.

The present study also demonstrates that the protection of BALB/c mice was associated with a significant decrease in the production of IL-4 and IL-10. Very low levels of parasite-specific IL-10 were detected after the stimulation of spleen cells derived from vaccinated mice, 10 weeks after infection. By contrast, spleen cells from both control mice showed a significantly higher production of this cytokine. Indeed, control of the parasite-mediated IL-10 response may be critical for protection, since this cytokine is considered to be the most important factor for VL progression after infection with viscerotropic *Leishmania* species in IL-10 deficient mice [74], [75], or in mice treated with an anti-IL-10 receptor antibody [76]. In BALB/c mice, the IL-4-dependent production of IgG1 antibodies is associated with disease progression into some *Leishmania* species, including *L. amazonensis* [77], and *L. infantum* [78]. Here, immunized mice with the phage clones presented higher levels of SLA-specific IgG2a antibodies, as compared to IgG1 levels, also demonstrating the development of a Th1 immune response in these animals.

Like observed to the IFN-γ and IL-12 levels, spleen cells from vaccinated mice, as compared to the control groups, produced higher levels of clones-specific GM-CSF, a cytokine related to macrophage activation and resistance in murine models against intracellular pathogens, such as *L. infantum* [77], *L. major* [79], and *L. donovani* [80]. It has also been reported that the administration of a therapeutic vaccine containing *Leishmania* antigens plus GM-CSF could be correlated with the curing of Tegumentary leishmaniasis patients [81].

In conclusion, the present study describes a subtractive phage display strategy that led to the identification of antigens to be evaluated as vaccines against *Leishmania* infection. The results indicated that two phage clones expressing target mimotopes conferred protection in BALB/c mice against *L. infantum*. Protection was correlated with CD4^+^ and, mainly, CD8^+^ T cells response, which was characterized by high levels of IFN-γ, IL-12, and GM-CSF, associated with low levels of IL-4 and IL-10. The data leads to the conclusion that the B10 and C01 phage clones may well be effective vaccine candidates against VL. Studies are in progress to identify and clone the native proteins that express these mimotopes, in an attempt to compare their efficacy with those found for mimotopes in the present study.

## Supporting Information

Figure S1 **Analysis of the cellular immune response.** The cellular response induced in the *in vitro* cultured spleen cells obtained from naive and *Leishmania infantum*-infected BALB/c mice was evaluated. For this, single cells suspensions of spleen cells were collected, pooled and *in vitro* cultured in 24-well plates (Nunc), at 5×10^6^ cells per mL, in duplicate. Cells were incubated in RPMI 1640 medium (Sigma; non-stimulated control), or separately stimulated with each phage clone (20 individual clones, with 1×10^10^ phages per well) for 48 h at 37°C, 5% CO_2_. The same experimental conditions were performed using spleen cells of naive mice. Wild-type and random non-specific phage clones were used as controls. The IFN-γ and IL-4 levels were determined in the culture supernatants, using commercial kits (BD OptEIA, Pharmingen), according to manufactureŕ instructions. In **A**, the results obtained using spleen cells of naive mice are showed. In **B**, the results obtained using spleen cells of *L. infantum-*infected mice are showed. Each bar represents the mean ± standard deviation (SD) of the cytokines levels. Experiments were repeated twice and presented similar results.
(TIF)
