# Supplementary figures and images for: Mimotope-Based Vaccines of Leishmania infantum Antigens and Their Protective Efficacy against Visceral Leishmaniasis
Source: PLoS One. 2014 Oct 15;9(10):e110014. doi: 10.1371/journal.pone.0110014 (PMC4198211; doi:10.1371/journal.pone.0110014)

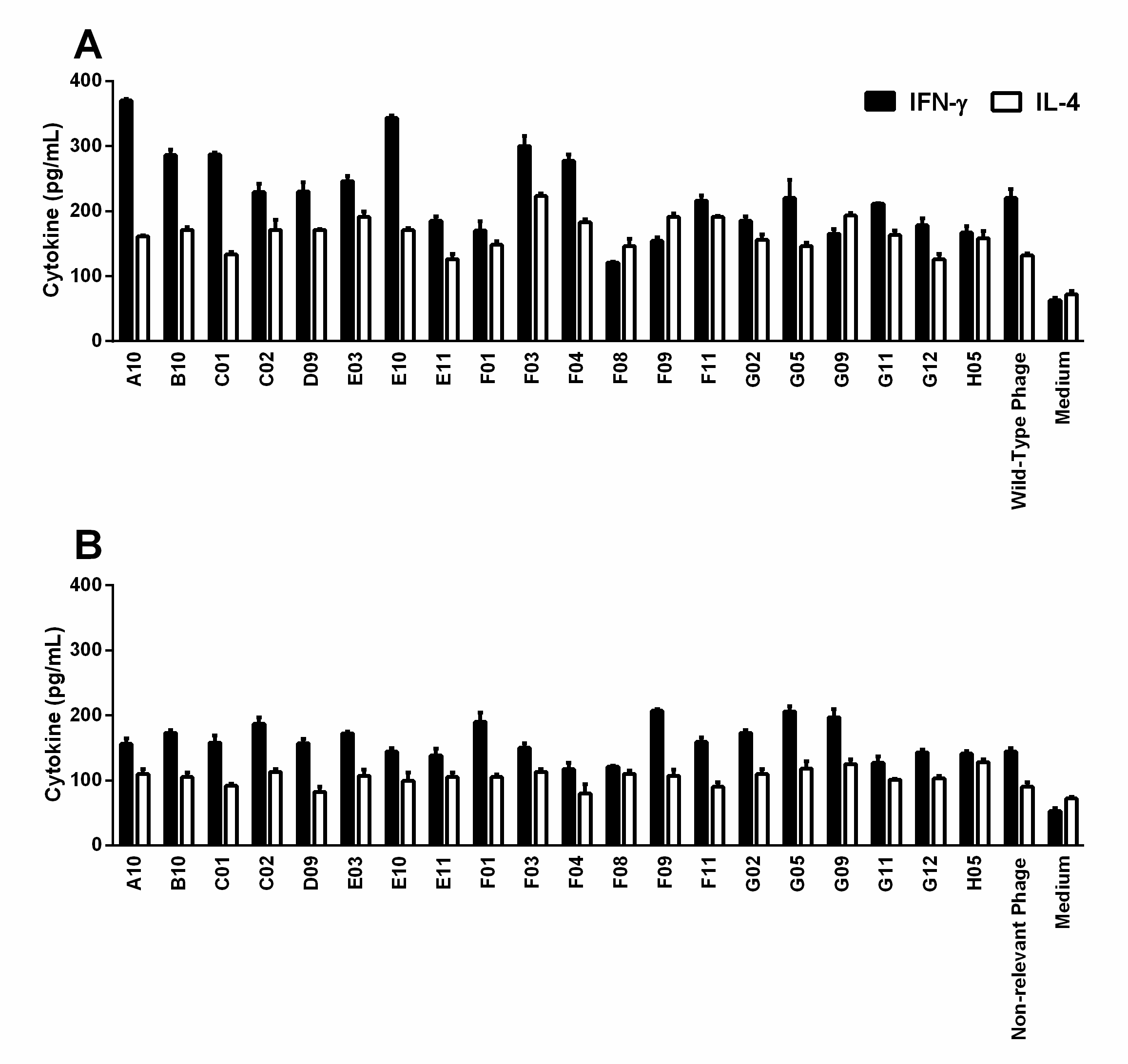

Supplement: Figure S1 — Analysis of the cellular immune response. The cellular response induced in the in vitro cultured spleen cells obtained from naive and Leishmania infantum-infected BALB/c mice was evaluated. For this, single cells suspensions of spleen cells were collected, pooled and in vitro cultured in 24-well plates (Nunc), at 5×10^6^ cells per mL, in duplicate. Cells were incubated in RPMI 1640 medium (Sigma; non-stimulated control), or separately stimulated with each phage clone (20 individual clones, with 1×10^10^ phages per well) for 48 h at 37°C, 5% CO2. The same experimental conditions were performed using spleen cells of naive mice. Wild-type and random non-specific phage clones were used as controls. The IFN-γ and IL-4 levels were determined in the culture supernatants, using commercial kits (BD OptEIA, Pharmingen), according to manufactureŕ instructions. In A, the results obtained using spleen cells of naive mice are showed. In B, the results obtained using spleen cells of L. infantum-infected mice are showed. Each bar represents the mean ± standard deviation (SD) of the cytokines levels. Experiments were repeated twice and presented similar results. (TIF) [file pone.0110014.s001.tif]
